# Detection of Minority Variants and Mixed Infections in Mycobacterium tuberculosis by Direct Whole-Genome Sequencing on Noncultured Specimens Using a Specific-DNA Capture Strategy

**DOI:** 10.1128/mSphere.00744-21

**Published:** 2021-12-15

**Authors:** Nuria Lozano, Val F. Lanza, Julia Suárez-González, Marta Herranz, Pedro J. Sola-Campoy, Cristina Rodríguez-Grande, Sergio Buenestado-Serrano, María Jesús Ruiz-Serrano, Griselda Tudó, Fernando Alcaide, Patricia Muñoz, Darío García de Viedma, Laura Pérez-Lago

**Affiliations:** a Instituto de Investigación Sanitaria Gregorio Marañón, Madrid, Spain; b Servicio de Microbiología Clínica y Enfermedades Infecciosas, Hospital General Universitario Gregorio Marañón, Madrid, Spain; c Bioinformatics Unit IRYCIS, University Hospital Ramón y Cajal, Madrid, Spain; d CIBER Enfermedades Infecciosas, Madrid, Spain; e Unidad de Genómica, Hospital General Universitario Gregorio Marañón, Madrid, Spain; f CIBER Enfermedades Respiratorias, CIBERES, Madrid, Spain; g Servei de Microbiologia, Hospital Clinic-CDB, Facultat de Medicina i Ciències de la Salut, Universitat de Barcelona, Barcelona, Spain; h Servicio de Microbiología, Hospital Universitario de Bellvitge-IDIBELL, L’Hospitalet de Llobregat, Barcelona, Spain; i Department of Pathology and Experimental Therapy, University of Barcelona, L’Hospitalet de Llobregat, Barcelona, Spain; j Departmento de Medicina, Universidad Complutense de Madrid, Madrid, Spain; Washington University School of Medicine in St. Louis

**Keywords:** *Mycobacterium tuberculosis*, whole-genome sequencing, heteroresistance, mixed infections, specific-DNA capture

## Abstract

Detection of mixed Mycobacterium tuberculosis (MTB) infections is essential, particularly when resistance mutations are present in minority bacterial populations that may affect patients’ disease evolution and treatment. Whole-genome sequencing (WGS) has extended the amount of key information available for the diagnosis of MTB infection, including the identification of mixed infections. Having genomic information at diagnosis for early intervention requires carrying out WGS directly on the clinical samples. However, few studies have been successful with this approach due to the low representation of MTB DNA in sputa. In this study, we evaluated the ability of a strategy based on specific MTB DNA enrichment by using a newly designed capture platform (MycoCap) to detect minority variants and mixed infections by WGS on controlled mixtures of MTB DNAs in a simulated sputum genetic background. A pilot study was carried out with 12 samples containing 98% of a DNA pool from sputa of patients without MTB infection and 2% of MTB DNA mixtures at different proportions. Our strategy allowed us to generate sequences with a quality equivalent to those obtained from culture: 62.5× depth coverage and 95% breadth coverage (for at least 20× reads). Assessment of minority variant detection was carried out by manual analysis and allowed us to identify heterozygous positions up to a 95:5 ratio. The strategy also automatically distinguished mixed infections up to a 90:10 proportion. Our strategy efficiently captures MTB DNA in a nonspecific genetic background, allows detection of minority variants and mixed infections, and is a promising tool for performing WGS directly on clinical samples.

**IMPORTANCE** We present a new strategy to identify mixed infections and minority variants in Mycobacterium tuberculosis by whole-genome sequencing. The objective of the strategy is the direct detection in patient sputum; in this way, minority populations of resistant strains can be identified at the time of diagnosis, facilitating identification of the most appropriate treatment for the patient from the first moment. For this, a platform for capturing M. tuberculosis-specific DNA was designed to enrich the clinical sample and obtain quality sequences.

## INTRODUCTION

Before the development of molecular techniques, Mycobacterium tuberculosis (MTB) infections were believed to be caused by a single clonal population (1). Complex infections with MTB (e.g., mixed infections), which can be identified by molecular genotyping techniques, have become an important challenge to the diagnosis, treatment, and control of tuberculosis (1–3).

Mixed infections can result from (i) simultaneous infection by different strains in the same patient or (ii) genomic evolution of a strain within the host and consequent coexistence of two populations. Both clonal heterogeneity events may involve strains with the same or different drug susceptibility; the latter is known as heteroresistance and is an increasing public health concern. Patients with undiagnosed heteroresistance receive suboptimal treatments; consequently, resistant bacterial populations may be selected due to antibiotic pressure, leading to poor patient outcomes (4). Detection of mixed infections is key even in the absence of heteroresistance, as they may be associated with a worse course of the disease, although the underlying reasons for this phenomenon are still unknown (5–7).

Detection of mixed infections and heteroresistance should be done as close to the diagnosis as possible, to prescribe the most appropriate treatment. Since MTB is a slow-growing microorganism, relevant diagnostic information should be obtained directly from the clinical sample. Different studies have shown that after culture, the representation of clonal variants of the sample decreases, and relevant information may be lost. Thus, noncultured clinical specimens are the optimal samples for studying mixed infections with MTB (8–10).

Mycobacterial interspersed repetitive unit–variable-number tandem repeats (MIRU-VNTR) is the classical genotyping molecular tool with the highest resolution and sensitivity for detecting mixed infections. However, it analyzes only 24 loci in the genome; therefore, certain mixed infections may not be detected, and heteroresistance resulting from microevolution goes unnoticed (1, 11). Whole-genome sequencing (WGS) allows detection of mixed infections or minority variants with high precision. Several studies have used WGS for the identification of mixed infections from bacterial cultures (1, 12, 13). Thus, to date, WGS may represent the best diagnostic alternative to detect mixed infections and heteroresistance directly from clinical samples. Unfortunately, there are many technical difficulties due to the presence of large amounts of nonspecific DNA in the samples from bacterial flora and human cells, which generally results in poor-quality sequences. Different approaches are increasingly being developed to optimize WGS from clinical samples, some with good results (8, 14, 15).

In this work, we explored a pilot approach for identifying mixed MTB infections and minority variants from clinical samples using WGS. A model of controlled artificial MTB DNA mixtures embedded in a genetic background from real sputa was used. To overcome the problem of large amounts of nonspecific DNA present in the sample, we designed a platform of MTB DNA capture probes (MycoCap) that allows specific enrichment of the sample with the MTB DNA and subsequently carried out WGS of the captured MTB DNA.

## RESULTS

**FIG 1:**
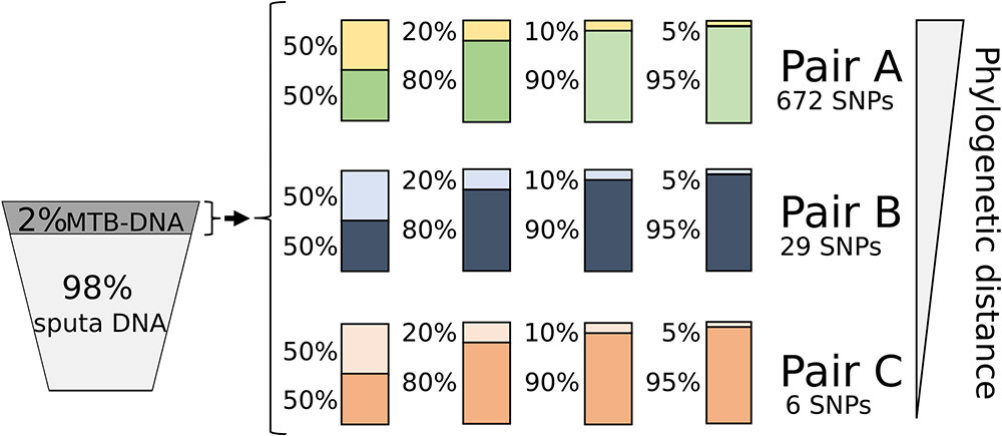
Representation of the composition of controlled mixtures subjected to the specific capture and sequencing strategy.

The aim of this study was to evaluate whether a strategy for specifically capturing MTB DNA can identify minority variants and mixed infections by WGS in a simulated sputum genetic background. The evaluation material consisted of artificial samples containing 98% sputum DNA and 2% controlled MTB DNA mixtures. Each mixture was prepared at four relative proportions (50:50, 80:20, 90:10, and 95:5) (Fig. 1) of different known sequences of MTB strains. Strains were combined to ensure three pairs with different phylogenetic distances between them (672, 29, and 6 single nucleotide polymorphisms [SNPs]) (Fig. 1). One of the pairs included a multidrug-resistant strain (pair A).

Our general strategy was divided into four specific stages: (i) MTB DNA captures plus WGS, (ii) identification of minority variants, (iii) detection of heteroresistance, and (iv) automatic detection of mixed infections.

### MTB DNA capture and whole-genome sequencing.

We performed the capture and subsequent WGS of MTB DNA from the 12 different artificial samples (Fig. 1). As controls, WGS was carried out from the same libraries without the capture step (Table 1).

**TABLE 1:** Results for the four capture sequencing quality parameters analyzed

Sample	% human DNA		% alignment with reference		Coverage depth		% coverage breadth (>20 reads)	
	Captured	Noncaptured	Captured	Noncaptured	Captured	Noncaptured	Captured	Noncaptured
1	4.24	80.91	90.22	2.3	70×	1.13×	95.05	0.04
2	4.44	79.57	89.77	2.39	73×	1.26×	94.4	0.04
3	4.82	79.97	89.16	2.22	86×	1.17×	96.14	0.04
4	4.96	79.63	88.63	2.04	85×	1.33×	96.92	0.05
5	4.95	90.54	89.11	2	59.6×	0.94×	93.1	0.04
6	5.84	80.07	87.34	1.8	57×	0.88×	91.17	0.05
7	5.27	81.08	88.65	1.73	48×	0.87×	89.04	0.04
8	4.48	78.82	89.54	2.36	99×	1.36×	96.84	0.05
9	4.9	79.89	89.04	2.06	81.9×	1.08×	96.4	0.05
10	4.16	79.05	89.94	2.48	167×	1.11×	98.51	0.04
11	4.32	81	90.35	2.08	66×	0.92×	94.98	0.04
12	3.23	78.39	91.87	2.86	76×	1.26×	96.22	0.04

We analyzed four parameters to evaluate the efficiency of the capture and quality of the sequences: (i) proportion of human reads, (ii) percentage of alignment with the MTB reference sequence, (iii) coverage depth, and (iv) breadth of coverage (Table 1).

The mean percentage of human reads in the sequences after the captures was 4.63%, compared to 80.7% when the same libraries were sequenced without the capture step (Table 1). The average percentage of alignment with the MTB reference sequence was around 90% for the captured set of samples, while the noncaptured samples showed an average of 2.19% (Table 1).

All samples in the capture set showed a coverage depth of >40×, with an average value of 62.5× (48× to 167×), while samples without capture were below 2× (Table 1). Captured samples showed a breadth of coverage around 95% for >20× reads. In contrast, noncaptured samples showed, at most, a genome coverage of 0.04% (for 20× reads).

### Identification of minority variants.

We focused on the positions where the strains included in each mixture showed allelic differences and performed a manual analysis by direct visualization of the sequences. Heterozygotes were detected in all proportions and for all pairs (Table 2). Ninety-nine percent of heterozygous calls were identified for the 50:50 and 80:20 ratios and almost 90% for the 90:10 ratio, for which 100% of the heterozygous positions were detected for pairs B and C. However, for the 95:5 ratio, a reduction of heterozygous calls was observed, being detected in 69% of the analyzed positions; only one heterozygous position was left to identify for pairs B and C.

**TABLE 2:** Number of heterozygous SNPs detected by the manual approach

Pair (no. of SNPs)	No. (%) of SNPs detected in mix			
	50:50	80:20	90:10	95:5
A (671)	670	667	586	452
B (29)	26	28	29	28
C (6)	5	6	6	5
Total (706)	701 (99.3)	701 (99.2)	621 (88)	485 (69)

Identification of an allelic variant in a 95:5 ratio could be considered a spurious call due to sequencing errors. To check if our 95:5 calls were robust, we evaluated if other calls at that proportion could occur in positions where heterozygosis calls were not expected. For this, we analyzed an equivalent number of homozygous positions located at 100 bp of each heterozygous position, ensuring the same genomic environment. Statistically significant differences were observed for all pairs; pair A, *P* < 2 × 10^−16^; pair B, *P* < 3 × 10^−10^; and pair C, *P* = 0.01671 (*P* < 0.05). Thus, the positions detected in the 95:5 ratio were correct calls.

### Detection of heteroresistance.

As pair A was constituted by a susceptible and a resistant strain, we evaluated whether identification of heteroresistance was possible in a nontargeted approach. Two independent nonexpert evaluators were asked to identify heteroresistance from a list with the most frequent resistant positions in the genome of MTB. They properly distinguished heteroresistance in the three resistance-associated variants (S531L, resistance to rifampicin; S315T, resistance to isoniazid; M306I, resistance to ethambutol) harbored by the MDR strain in 50:50, 80:20, and 90:10 ratios. However, they did not identify the resistant mutations in the 95:5 proportion.

### Automatic detection of mixed infections.

**FIG 2:**
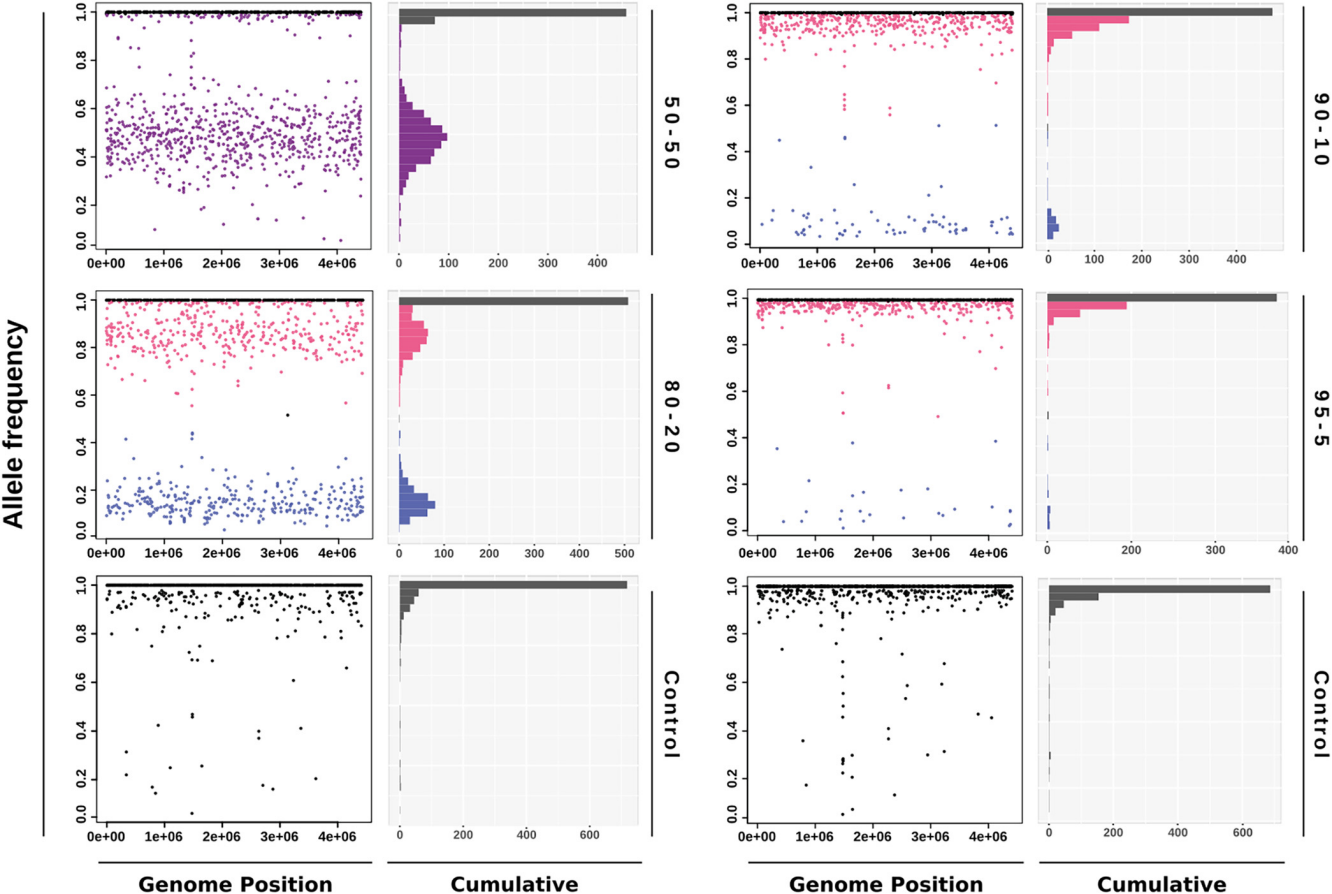
Graphic representation of allelic frequency distribution of the high-quality differential single nucleotide polymorphisms for each strain in pair A. In each pair, the left panel shows the allelic frequency distribution along the genome and the right panel shows the cumulative allelic frequency distribution. Controls were homozygous strains subjected to the same specific-DNA capture and WGS approach as the controlled mixtures.

Taking advantage of the combination of two different strains in pair A, we evaluated whether mixed infections could be identified by using an automatic approach that provides, based on the LoFreq results, the differential distribution of allelic frequency of specific high-quality SNPs for each strain in the mixture. Mixed infection was clearly detected visually in the 50:50, 80:20, and 90:10 ratios (Fig. 2). An accumulation of heterozygous SNPs was seen at position 0.5 for the 50:50 ratio, while the segregation of two populations was differentiated in the 80:20 and 90:10 ratios.

## DISCUSSION

Genomics is undeniably the most accurate tool with the highest resolution power for the study and analysis of MTB at both diagnostic and epidemiological levels (16–19). MTB is a slow-growing microorganism, and it takes 2 to 3 weeks to achieve results from cultures; thus, it is key to perform WGS directly from the clinical sample. Furthermore, cultures of MTB samples may cause loss of clonal diversity, mainly of minority variants present in the clinical sample, and relevant information is therefore lost (8–10). Loss of variability is especially relevant in the case of heteroresistance, as it may lead to wrong treatments and poor outcomes (20).

WGS directly from clinical samples is a great current scientific challenge due to the presence of large amounts of accompanying DNA from the flora of the respiratory tract and human cells that interfere with this sequencing tool. Different approaches have been tested to try to overcome these interferences, such as selectively eliminating the human DNA present in the clinical sample (14) or using MTB DNA enrichment systems, e.g., specific biotinylated RNA baits (4, 8, 15, 21, 22).

In our study, an MTB DNA enrichment strategy was applied, but unlike other authors, we used an in-house DNA capture platform based on total MTB pangenome sequences (MycoCap). RNA baits are often used as a specific DNA enrichment strategy. The two systems have in common the use of probes to first hybridize and then capture the specific DNA through magnetic beads or the biotin-streptavidin duo. The performance of RNA and DNA bait systems is mostly equivalent; the differences in capture efficiency between the two approaches lie mainly in the design of the probes and the characteristics of the genome to be captured. Zhou et al. (23) concluded that although, in general terms, double-stranded RNA baits are the most optimal strategy, for DNAs with a high GC content, as happens in MTB, DNA baits provide better performance. We based our MycoCap platform on a more refined design than the standard approaches, which use a single reference sequence. We generated DNA probes covering the MTB pangenome, defined from hundreds of MTB genomes available in public databases. To test the effectiveness of our strategy, we carried out WGS on 12 samples containing purified DNA (2% MTB DNA and 98% DNA from sputa) in order to simulate the DNA combination present in specimens from patients with medium- to high-load MTB infections.

The use of an MTB DNA capture step with the MycoCap platform was definitive. It resulted in sequences with the same quality as those obtained from culture. However, the same libraries sequenced without the capture step could not be analyzed because they did not meet the minimum established quality criteria. It is an all-or-nothing result; the capture step was key to carry out the analyses of the sequences.

Reading depth values and average amplitude coverage were similar to those reported in other studies that used specific DNA enrichment techniques from MTB (8, 15) and gave better results than those obtained after the optimization strategy of the DNA extraction without sample enrichment (14).

In the above-mentioned studies, WGS directly from clinical samples allowed detection of resistance mutations in most sequenced cases with high concordance with the results from sequences after culture and phenotypic tests. In addition, in the study carried out by Nimmo et al. (8), greater genomic diversity was detected in clinical samples than in isolates from cultures. Goig et al. (15) used for the first time sequences obtained by WGS from clinical samples to carry out epidemiological analysis, integrating them with 780 sequences of strains circulating among the population.

The good results obtained in the specific capture of MTB DNA under conditions of a nonspecific genetic background gave us the opportunity to evaluate the ability of our strategy (DNA capture and WGS) to detect minority populations in clinical samples. Our experimental design, based on controlled mixtures, allowed us to determinate its efficacy for identifying interstrain heterozygosis in the positions involving SNPs.

The manually targeted analysis of the known heterozygous positions in the different proportions of mixed samples detected minority variants in almost 90% of the positions where they existed. As expected, the lowest percentage of heterozygous calls was obtained for the 95:5 ratio; their robustness was analyzed and sequencing errors were ruled out. Despite this, for minority variants below 10%, heterozygous calls should be considered robust only when the corresponding positions have a sequencing coverage depth of >60× (which would correspond to 3 reads at a 95:5 ratio).

The directed manual approach allowed the detection of minority variants up to a ratio of 95:5 in 69% of the heterozygous positions and close to 90% in the 90:10 ratio; the blind participants correctly identified the resistance mutations when they were present in 10% of the population. We must acknowledge that the visual inspection required in the manual approach cannot be systematically applied to a real diagnostic setting. However, it might still find an analytical niche when the presence of heteroresistant subpopulations is expected, and therefore, the inspection of the positions for the most frequent resistance mutations is feasible. Detection of resistant populations at a proportion of 10% improves the detection obtained by the most widely used molecular tests, GeneXpert MTB/RIF and Xpert MTB/RIF Ultra, which identify rifampicin-resistant mutations only when the proportion of resistant strains is between 20 and 80% (24–26). Some molecular techniques allow detecting heteroresistance up to a 95:5 ratio (such as GenoType MTBDRplus or deep amplicon sequencing) (24–30), but these approaches are limited to the analysis of a previous selection of loci. In contrast, our proposal can target any loci along the chromosome.

Once the ability to capture and detect minority populations using this strategy was demonstrated, we focused on analyzing the ability of the strategy to automatically identify mixed infections. It was possible to clearly detect mixed infections up to a 90:10 ratio. This is in line with results reported by other authors who identified mixed infections using WGS when the minority population was above 10% using *in silico* and *in vitro* artificial samples (12, 30). In our case, the MTB DNA was mixed with 98% of nonspecific DNA.

Although the automatic approach cannot detect mixtures between close clonal variants due to the low number of differential SNPs between the strains, it does allow visualization of all heterozygous positions in the resulting variant call files from the LoFreq analysis. Mixed infections, in this case, could be detectable if the proportion of both strains was similar (between 40 and 60%). Subsequently, the directed manual approach can be applied for detailed in-depth analysis and confirming/ruling out heterozygosity. This dual strategy may be useful in case of suspected mixed infection due to the circulation of prevalent endemic strains in certain populations with high incidence or for patients with a long diagnostic delay.

Only the introduction of a specific MTB DNA capture step prior to WGS made it possible to carry out the different analyses presented in this study. Our strategy offers sequencing quality parameters similar to those obtained when sequencing pure MTB cultures, even in circumstances where MTB is severely underrepresented in a non-MTB sputum DNA background. This DNA capture WGS approach allows the automatic detection of mixed infections and identification of minority variants in known positions, including the detection of heteroresistance. The same samples could not have been analyzed without the capture step, so the capture platform (MycoCap) designed for this study is a promising tool to open the path to perform WGS directly on clinical samples for diagnostic or epidemiological purposes.

## MATERIAL AND METHODS

### Generation of a DNA pool from sputa.

We selected 15 anonymized sputa from different patients with suspected MTB infection, which was ruled out by a negative result after 42 days of incubation in a mycobacterial growth indicator tube (MGIT; Becton Dickinson, New Jersey, USA). DNA was extracted from 1 ml of each decontaminated sediment using minikit DNA (Qiagen, Hilden, Germany) following the manufacturer’s instructions. We mixed all the DNAs, and the generated pool was quantified using a Quantus fluorometer (Promega, Madison, WI, USA). A concentration of 72 ng/ml was obtained. This pool was used as a base to prepare control mixed samples.

### MTB DNA/sputum DNA controlled mixtures.

Twelve artificial DNA samples were prepared, each containing 72 ng of DNA composed of 98% of the DNA pool from the sputa and 2% of the MTB DNA mixture (Fig. 1). The purified DNA of each sample was quantified using the Quantus fluorometer (Promega). We considered a MTB genome size of 4.4 Mb and assumed that 10 fg corresponds to two genome equivalents (31). Based on these values, we calculated the amount of MTB DNA to be included in the mixtures to simulate a sputum load of around 10^5^ CFU/ml (within the smear-positive range).

The 2% of MTB DNA of each sample was composed of DNA mixtures from two MTB strains of known sequence in different proportions: 50:50, 80:20, 90:10, and 95:5 (Fig. 1). We prepared three pairs of strains with different phylogenetic distances. For pair A, DNAs were from completely different strains (672 SNPs between them); the minority DNA came from a multidrug-resistant (MDR) strain, while the majority DNA was from a susceptible strain. For pair B, DNAs were from intermediate related strains (29 SNPs between them), and for pair C, DNAs were from closely related strains (six SNPs between them). Strains of pair B were drug susceptible, and strains of pair C were MDR.

### Library preparation and DNA capture of MTB DNA.

Each MTB DNA–sputum DNA mixture was fragmented by sonication using a Bioruptor Sonicator (Diagenode, Liège, Belgium), 20 kHz, with 60 cycles of 30 s on and 30 s off. Libraries were prepared with the Kapa Hyperprep kit, following the manufacturer’s instructions (Roche, Basel, Switzerland), and the quality of the libraries produced was checked with the LabChip (PerkinElmer, Massachusetts, USA) instrument. Next, the 12 libraries were pooled in equimolecular amounts and subjected to targeted sequence capture with an MTB-specific DNA capture platform (MycoCap). MycoCap is based on Roche-NimbleGen’s SeqCapEZ technology (currently HyperCap; Roche-NimbleGen, Madison WI, USA) (see Fig. S1 in the supplemental material). This technology is optimized for the enrichment of previously designed DNA regions. For the design of MycoCap, 3,649 genomes from RefSeq-NCBI were used. A pangenome of 132,885 nonredundant genes was built, clustered for 99% identity and 80% coverage. This ensured a sufficient tiling to cover the great majority of the genes present in any strain of M. tuberculosis. The protocol for using MycoCap is the same as recommended by Roche-NimbleGen in any of their SeqCapEZ designs. Briefly, we prepared the hybridization using vacuum centrifugation; we mixed 1 μg of the library pool with 10 μl of the SeqCap EZ Developer reagent and 5 μl of HyperCap universal blocking oligonucleotides and concentrated the mixture in a SpeedVac for 30 min at 60°C. The library pool was incubated with 4 μl of the MycoCap probes for 5 min at 95°C and 20 h at 47°C. Finally, it was washed and recovered using the HyperCap target enrichment and HyperCap bead kits. Captured libraries were sequenced in a MiSeq device (Illumina, San Diego, CA, USA). As a control, the same libraries without the capture step were sequenced under identical conditions.

10.1128/mSphere.00744-21.1 FIG S1 MycoCap platform design and the capture and sequencing strategy. Download FIG S1, TIF file, 0.3 MB. Copyright © 2021 Lozano et al. 2021 Lozano et al. https://creativecommons.org/licenses/by/4.0/ This content is distributed under the terms of the Creative Commons Attribution 4.0 International license.

### Bioinformatics analysis.

The Fastq files were aligned using Burrows-Wheeler Aligner (BWA; v0.7.17-r1188). Variant calling was done by LoFreq (v2.1.3.1) and GATK (v4.0.6.0) for data preprocessing, following GATK’s best practices protocol. Next, repetitive sequences in the MTB genome (PE/PPE proteins, phage and repeat sequences) were filtered with a homemade script. SNPs within high-density zones were eliminated, by an in-house script, allowing a maximum of two SNPs in a 20-bp-width sliding window to avoid false SNPs derived from sequencing errors.

The Integrative Genomics Viewer (IGV) program was used for manual validation of the strategy and in the blind assay to detect heteroresistance, to visualize the percentage of calls from each mixed-DNA pair. For the blind assay, we provided two nonexpert revisers with a list including the most frequent MTB mutations conferring resistance to first- and second-line drugs and the .bam files with the different proportions of pair A.

In the automatic approach, to give greater robustness to the detected heterozygous positions, we filtered the SNPs called by the LoFreq program (32) with a database of SNPs from sequences of more than 500 strains and kept only the SNPs that had been previously described.

Histogram graphs were constructed with the aid of the R programming language and the specialized package tidyverse.

### Statistical analysis.

The Wilcoxon test was used to compare the percentage of minority variants identified in heterozygous positions with respect to the percentage of variants generated by sequencing errors identified in homozygous positions in the 95:5 proportion of the three analyzed pairs.

### Ethical considerations.

The study was approved by the ethics committee of Hospital General Universitario Gregorio Marañón (MICRO.HGUGM_326/18). For ethical reasons, reads of human sequences were detected and discarded by DeconSeq (v. standalone 0.4.3) against Human Genome Assembly GRCh38.p7.

### Data availability.

The fastq files were deposited in ENA (https://www.ebi.ac.uk); the accession number of the sequenced strains used in the mixes is PRJEB46134, and that of the resulting sequencing of the artificial mixes is PRJEB46132.
