# Supplementary figures and images for: Detection of Minority Variants and Mixed Infections in Mycobacterium tuberculosis by Direct Whole-Genome Sequencing on Noncultured Specimens Using a Specific-DNA Capture Strategy
Source: mSphere. 2021 Dec 15;6(6):e00744-21. doi: 10.1128/mSphere.00744-21 (PMC8673255; doi:10.1128/mSphere.00744-21)

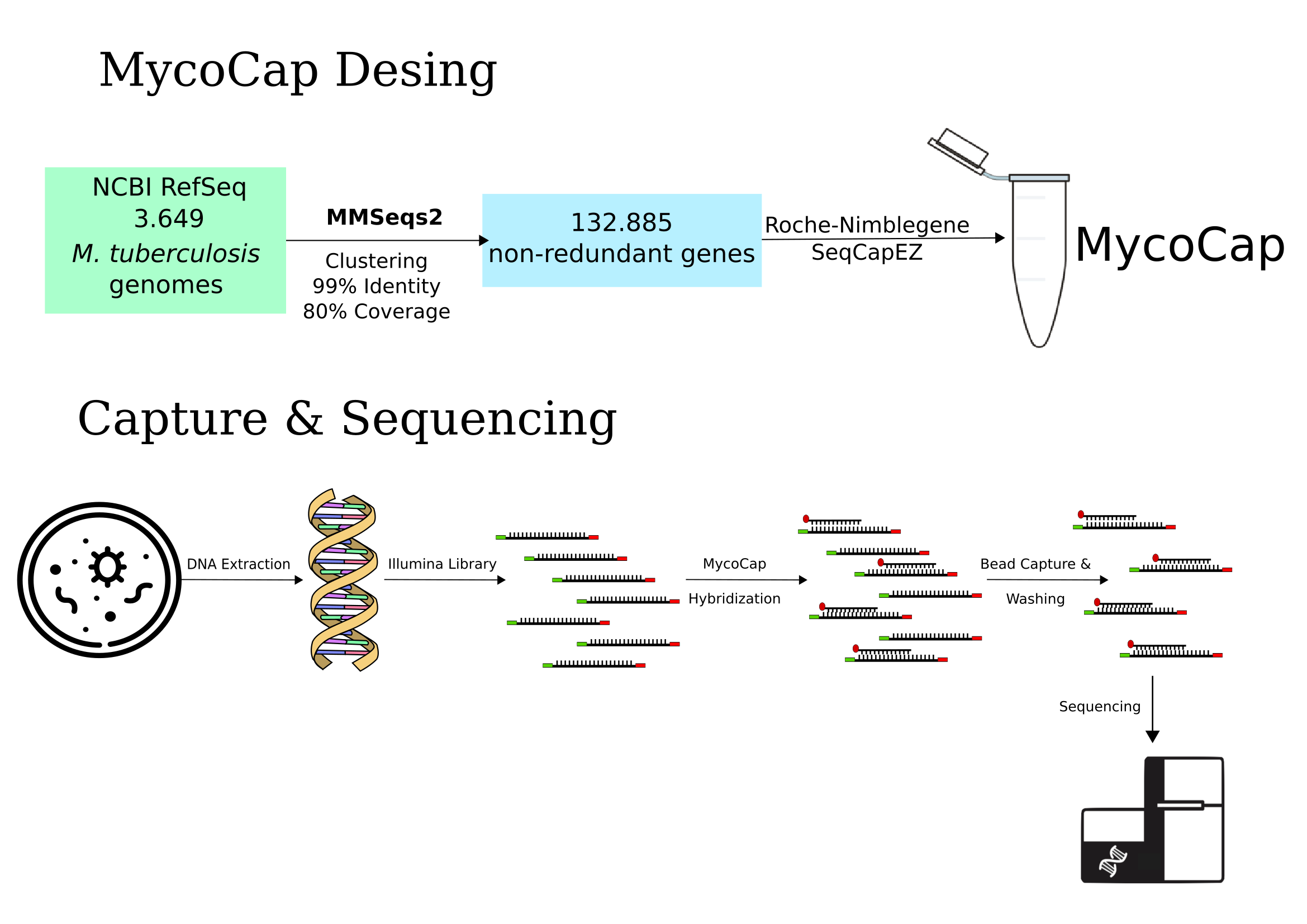

Supplement: FIG S1 [file msphere.00744-21-sf001.tif]
